# Intrathecal, Polyspecific Antiviral Immune Response in Oligoclonal Band Negative Multiple Sclerosis

**DOI:** 10.1371/journal.pone.0040431

**Published:** 2012-07-09

**Authors:** Isabel Brecht, Benedikt Weissbrich, Julia Braun, Klaus Viktor Toyka, Andreas Weishaupt, Mathias Buttmann

**Affiliations:** 1 Department of Neurology, University of Würzburg, Würzburg, Germany; 2 Institute of Virology and Immunobiology, University of Würzburg, Würzburg, Germany; Institute Biomedical Research August Pi Sunyer (IDIBAPS) - Hospital Clinic of Barcelona, Spain

## Abstract

**Background:**

Oligoclonal bands (OCB) are detected in the cerebrospinal fluid (CSF) in more than 95% of patients with multiple sclerosis (MS) in the Western hemisphere. Here we evaluated the intrathecal, polyspecific antiviral immune response as a potential diagnostic CSF marker for OCB-negative MS patients.

**Methodology/Principal Findings:**

We tested 46 OCB-negative German patients with paraclinically well defined, definite MS. Sixteen OCB-negative patients with a clear diagnosis of other autoimmune CNS disorders and 37 neurological patients without evidence for autoimmune CNS inflammation served as control groups. Antibodies against measles, rubella, varicella zoster and herpes simplex virus in paired serum and CSF samples were determined by ELISA, and virus-specific immunoglobulin G antibody indices were calculated. An intrathecal antibody synthesis against at least one neurotropic virus was detected in 8 of 26 (31%) patients with relapsing-remitting MS, 8 of 12 (67%) with secondary progressive MS and 5 of 8 (63%) with primary progressive MS, in 3 of 16 (19%) CNS autoimmune and 3 of 37 (8%) non-autoimmune control patients. Antibody synthesis against two or more viruses was found in 11 of 46 (24%) MS patients but in neither of the two control groups. On average, MS patients with a positive antiviral immune response were older and had a longer disease duration than those without.

**Conclusion:**

Determination of the intrathecal, polyspecific antiviral immune response may allow to establish a CSF-supported diagnosis of MS in OCB-negative patients when two or more of the four virus antibody indices are elevated.

## Introduction

Cerebrospinal fluid specific oligoclonal bands (OCB) were shown to be present in more than 95% of patients with clinically definite multiple sclerosis (MS) in Western populations [1]. They were part of the so-called McDonald diagnostic criteria for patients with relapsing-remitting MS (RRMS) until recently, when the international expert panel eliminated CSF examination as an essential part of the diagnostic work-up. Abnormal CSF findings are still part of the formal criteria for the diagnosis of primary progressive MS (PPMS) [2]. Absence of OCB in the CSF should enhance awareness of an alternative diagnosis.

It has long been known that about 90% of MS patients show intrathecal synthesis of antibodies against one or more neurotropic viruses [1], [3]. While detected slightly less frequently than OCB in MS patients, this antiviral immune response has demonstrated higher specificity for MS than OCB which may be present in a number of chronic inflammatory CNS conditions that can mimic MS. In contrast, intrathecal antiviral antibody synthesis is only rarely observed in patients with neuromyelitis optica (NMO), paraneoplastic neurological syndromes, neuroborreliosis, and tropical spastic paraparesis [1], [4]–[9]. Based on our anecdotal clinical observations of an intrathecal antiviral immune response in OCB-negative MS patients, we systematically evaluated the antiviral immune response in a cohort of well-defined MS patients, where no OCB were detected in the CSF.

## Methods

### Ethics Statement

Our study was approved by the Ethics Committee of the Faculty of Medicine at the University of Würzburg. All lumbar punctures were performed for diagnostic reasons with written informed consent from all patients, including usage of their CSF and serum samples for research purposes.

**Table 1:** Characteristics of CNS autoimmune disease control group.

Patient no.	Diagnosis	Age	Sex	Disease duration	Autoantibodies or CNS histology	Malignancy
1	Cerebellitis	52	Male	3 years	Anti-Hu	No
2	Cerebellitis	71	Male	2 months	Anti-NMDA IgM	No
3	CNS lupus erythematosus	68	Female	3 years	Antinuclear antibodies, anti-doublestranded DNA	No
4	Limbic encephalitis	65	Female	2 weeks	Anti-Yo	Cervix carcinoma
5	Limbic encephalitis	24	Female	3 years	Anti-glutamic acid decarboxylase	Papillary thyroid carcinoma
6	Longitudinally extensivetransverse myelitis	51	Female	6 days	Anti-aquaporin-4	No
7	Neuromyelitis optica	57	Female	8 years	Anti-aquaporin-4, anti-NMDA IgG	No
8	Neuromyelitis optica	53	Female	1 year	Anti-aquaporin-4	No
9	Neuromyelitis optica	49	Female	7 years	Anti-aquaporin-4	No
10	Neuromyelitis optica	40	Female	6 years	Anti-aquaporin-4	Breast cancer
11	Neuromyelitis optica	30	Female	6 months	Anti-aquaporin-4	No
12	Neuromyelitis optica	48	Female	3 years	Anti-aquaporin-4	No
13	CNS sarcoidosis	57	Female	10 months	Autopsy	No
14	CNS sarcoidosis	28	Male	14 months	Brain biopsy	No
15	Stiff person syndrome	62	Female	10 years	Anti-glutamic acid decarboxylase	No
16	Stiff person syndrome	52	Female	4 months	Anti-amphiphysin	Breast cancer

### Patients

Having treated several thousand MS patients at our department over the last years, an electronic database search revealed 46 patients of Caucasian origin between 2004 and 2010 with clinically definite MS, in whom a CSF analysis had shown less than 2 CSF-restricted bands on isoelectric focusing followed by an IgG immunoblot assay (Helena Biosciences via Sekisui Virotech GmbH, Rüsselsheim, Germany) and, in addition, the immunoglobulin G (IgG) index had been normal (defined as [CSF/serum IgG] : [CSF/serum albumin] ≤0.7) [10]. Aiming at high diagnostic specificity for MS all patients additionally fulfilled the following criteria: 1) MRI dissemination in space according to the 2005 McDonald criteria [11]; 2) unequivocal evidence for demyelination, as revealed by visual, magnetic motor and/or somatosensory evoked potentials; 3) negative differential diagnostic work-up according to the consensus report by Miller et al. [12]. In 8 of the 20 chronic progressive patients, a careful evaluation of patient histories did not reveal evidence for even single bouts of neurological symptoms, who were therefore classified as PPMS. Twelve of the 20 chronic progressive patients fulfilled the criteria for secondary progressive MS (SPMS). Overall, disease duration in MS patients was 1–40 years with a median of 8 years.

As controls we investigated two groups of patients: 1) 37 OCB-negative patients had other neurological disorders with no evidence for autoimmune CNS inflammation (migraine [n = 16], idiopathic peripheral facial palsy [n = 12], idiopathic intracranial hypertension [n = 7], non-inflammatory polyneuropathy [n = 1], subarachnoid hemorrhage [n = 1]). 2) Sixteen OCB-negative patients had autoimmune disorders of the CNS where the clinical syndrome in conjunction with detection of specific serum autoantibodies or CNS histology, and a careful differential diagnostic work-up allowed to exclude MS and to provide a specific diagnosis (Table 1). A comparison of demographic variables and routine CSF findings between both control groups and MS patients demonstrated some imbalance between the groups (Table 2).

**Table 2:** Comparison of demographic variables and CSF findings between control groups and MS patients.

	NCAND	CAND	MS total	RRMS	SPMS	PPMS
**Number of patients**	37	16	46	26	12	8
**Age at time of spinal tap, years** **(median [range])**	38 [16–78]	52 [24–71]	45 [19–69]	41 [19–59]	46 [30–68]	55 [46–69]
**Sex (men:women)**	1∶1.6	1∶4.3	1∶1.4	1∶1.8	1∶2.0	1∶0.3
**Age at onset, years** **(median [range])**		50 [20–71]	34 [12–57]	34 [12–57]	32 [17–45]	41 [28–56]
**Disease duration, years (median [range])**		2 [0–10]	8 [1–40]	3 [1–28]	14 [4–40]	12 [4–30]
**EDSS (median [range])**			3.5 [1]–[9]	2 [1–6.5]	6 [2]–[9]	5 [1.5–9]
**CSF cell count >4/µl**	1/37 [3%]	6/16 [42%]	5/46 [11%]	3/26 [12%]	2/12 [17%]	0/8 [0%]
**CSF cells/µl (median [range])**	1 [1]–[6]	4 [1–29]	2 [1–20]	2 [1–20]	2 [1]–[13]	1 [1]–[4]
**CSF Q_alb_, elevated (age adjustment according to ** [**16**] **)**	8/37 [22%]	12/16 [75%]	17/46 [37%]	9/26 [35%]	5/12 [42%]	3/8 [38%]

Abbreviations: NCAND, Non-CNS-autoimmune neurological disorders; CAND, CNS-autoimmune neurological disorders; EDSS, Expanded Disability Status Scale; Q_alb_, albumin CSF/serum ratio; MS, multiple sclerosis; RRMS, relapsing-remitting MS; SPMS, secondary progressive MS; PPMS, primary progressive MS.

### CSF Investigations

All routine CSF analyses were performed by the certified CSF laboratory at the Department of Neurology (A.W., K.V.T.) and included isoelectric focusing of CSF and serum. Intrathecal IgG synthesis against measles (M), rubella (R), varicella zoster (Z) and herpes simples (H) virus was retrospectively determined in all patients from paired serum and CSF samples, stored at −20°C, at the Department of Virology (J.B., B.W.) in a blinded fashion. Antiviral antibody concentrations were measured by commercial ELISA kits (Enzygnost®, Siemens Healthcare Diagnostics, Eschborn, Germany), essentially as described previously [13]. Intrathecal synthesis was determined by the antibody index (AI) method [3]. In brief, AI values were calculated using the formula:

AI = (IgG_spec [CSF]_/IgG_spec [serum]_) : (IgG_total [CSF]_/IgG_total[serum]_) = Q_spec_/Q_IgG_, where spec indicates the specific virus to which the antibodies were tested as part of the total IgG concentration in serum and CSF. Q_IgG_ was replaced by Q_lim_ if Q_IgG_ > Q_lim_, as suggested by Reiber and Lange [3]. Q_lim_ represents the upper limit of the Q_IgG_ under the assumption that the IgG fraction in the CSF originates only from blood. Q_lim_ can be calculated for an individual patient from the CSF/serum quotient of albumin (Q_Alb_). AI values ≥1.5 were considered positive [3]. Inter-assay reproducibility of the Q_spec_ determinations was as follows: M 15.4%, R 17.3%, Z 18.9%, H 18.3%.

### Statistical Analysis

Values of AIs, age and disease duration were compared between groups by Kruskal-Wallis analysis, followed by Dunn’s multiple comparison test. Age at onset between SPMS and PPMS patients was compared by the Mann*-*Whitney U test. Fisher’s exact test was employed to compare frequencies of elevated AIs in SPMS and PPMS patients and AI positivity in patients with a disease duration of less vs. more than 5 years. All tests were two-tailed. P values of <0.05 were defined as statistically significant. All calculations were performed with GraphPad Prism 4 software (La Jolla, CA).

**Table 3:** Frequencies of mono-, bi-, tri- and quadrispecific intrathecal antiviral immune responses in control and MS patients.

Antibody indices	NCAND	CAND	MS total	RRMS	SPMS	PPMS
**quadrispecific**	0/37 [0%]	0/16 [0%]	1/46 [2%]	0/26 [0%]	1/12 [8%]	0/8 [0%]
**trispecific**	0/37 [0%]	0/16 [0%]	2/46 [4%]	0/26 [0%]	2/12 [17%]	0/8 [0%]
**bispecific**	0/37 [0%]	0/16 [0%]	8/46 [17%]	2/26 [8%]	5/12 [42%]	1/8 [13%]
**monospecific**	3/37 [8%]	3/16 [19%]	10/46 [22%]	6/26 [23%]	0/12 [0%]	4/8 [50%]
**negative**	34/37 [92%]	13/16 [81%]	25/46 [54%]	18/26 [69%]	4/12 [33%]	3/8 [38%]

Mono, bi-, tri- and quadrispecific denote number of elevated virus-specific antibody indices identified in any individual patient. Note that percent values may not add up to 100 due to arithmetic rounding. Abbreviations: see Table 2.

**Table 4:** Combinations of intrathecal antibody production in control patients and patients with MS subtypes.

AI combination	NCAND	CAND	RRMS	SPMS	PPMS
**M+ R+ Z+ H+**	0/37 [0%]	0/16 [0%]	0/26 [0%]	1/12 [8%]	0/8 [0%]
**M+ R+ Z+**	0/37 [0%]	0/16 [0%]	0/26 [0%]	2/12 [17%]	0/8 [0%]
**M+ R+ H+**	0/37 [0%]	0/16 [0%]	0/26 [0%]	0/12 [0%]	0/8 [0%]
**M+ Z+ H+**	0/37 [0%]	0/16 [0%]	0/26 [0%]	0/12 [0%]	0/8 [0%]
**R+ Z+ H+**	0/37 [0%]	0/16 [0%]	0/26 [0%]	0/12 [0%]	0/8 [0%]
**M+ R+**	0/37 [0%]	0/16 [0%]	1/26 [4%]	1/12 [8%]	0/8 [0%]
**M+ Z+**	0/37 [0%]	0/16 [0%]	0/26 [0%]	2/12 [17%]	1/8 [13%]
**M+ H+**	0/37 [0%]	0/16 [0%]	1/26 [4%]	0/12 [0%]	0/8 [0%]
**R+ Z+**	0/37 [0%]	0/16 [0%]	0/26 [0%]	2/12 [17%]	0/8 [0%]
**R+ H+**	0/37 [0%]	0/16 [0%]	0/26 [0%]	0/12 [0%]	0/8 [0%]
**Z+ H+**	0/37 [0%]	0/16 [0%]	0/26 [0%]	0/12 [0%]	0/8 [0%]
**M+**	0/37 [0%]	3/16 [19%]	1/26 [4%]	0/12 [0%]	2/8 [25%]
**R+**	0/37 [0%]	0/16 [0%]	3/26 [12%]	0/12 [0%]	1/8 [13%]
**Z+**	3/37 [8%]	0/16 [0%]	1/26 [4%]	0/12 [0%]	1/8 [13%]
**H+**	0/37 [0%]	0/16 [0%]	1/26 [4%]	0/12 [0%]	0/8 [0%]
**M- R- Z- H-**	34/37 [92%]	13/16 [81%]	18/26 [69%]	4/12 [33%]	3/8 [38%]

Note that percent values may not add up to 100 due to arithmetic rounding. Abbreviations: M, measles virus; R, rubella virus; Z, varicella zoster virus; H, herpes simplex virus; see also Table 2.

## Results

An intrathecal antiviral immune response against at least one neurotropic virus was detected in 8 of 26 (31%) of the RRMS, 8 of 12 (67%) of the SPMS and 5 of 8 (63%) of the PPMS patients. In 3 of 16 (19%) of the CNS autoimmune (patients no. 6, 7 and 11 in Table 1) and in 3 of 37 (8%) of the non-autoimmune control patients (idiopathic intracranial hypertension [n = 3]) we found an elevated AI to any single virus. Elevated AIs against two or more viruses were detected in 2 of 26 (8%) of the RRMS, 8 of 12 (67%) of the SPMS, 1 of 8 (13%) of the PPMS and none of the control patients (Table 3). While the overall prevalence of a detectable antiviral immune response did not differ significantly between PPMS and SPMS patients, SPMS patients showed reactivity against at least two viruses significantly more often (p = 0.02). Details on the detected AI combinations are provided in Table 4.

**Figure 1:**
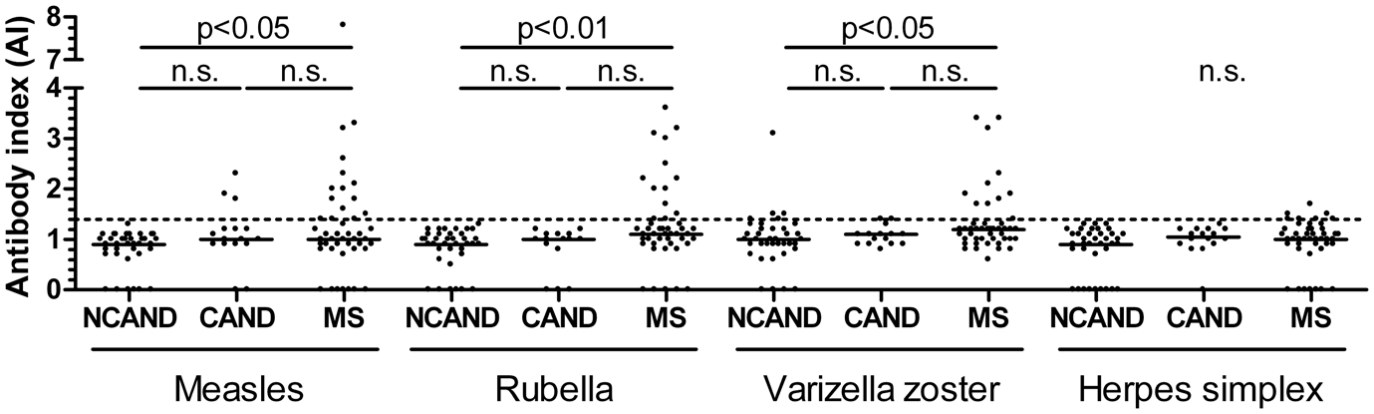
Antibody indices (AI) for the indicated viruses in control and MS patients. Dots represent individual values, lines reflect median values. AI was defined as 0, when virus antibodies were not detectable (n = 50 of 396). The dotted line reflects the upper limit of the normal range (1.4 [3]). Statistics: Kruskal-Wallis test for each virus AI, followed by Dunn’s multiple comparison test. P values of <0.05 were defined as statistically significant. Abbreviations: see Table 2.

Median AI values were significantly higher in MS than in non-CNS autoimmune control patients for measles, rubella and zoster viruses, but did not differ significantly from the smaller autoimmune control group (Figure 1). The NCAND patient with a varicella zoster AI of 3.1 was 39 years of age and had a history of chicken pox as a 9-year-old, but no history of shingles.

**Figure 2:**
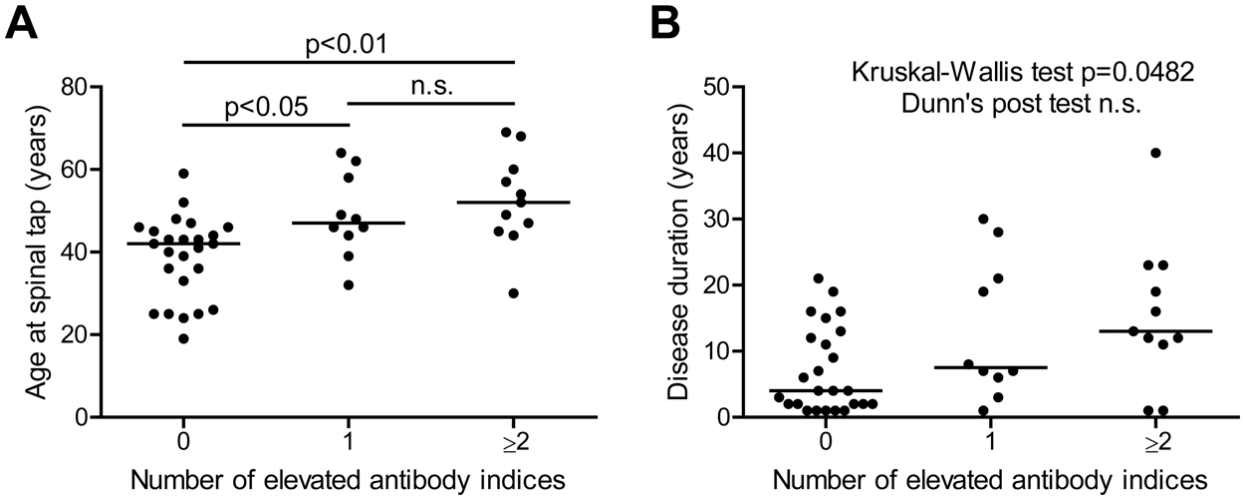
Correlation of age and disease duration with the intrathecal antiviral immune response. Multiple sclerosis patients (n = 46) were grouped according to the number of elevated AIs and analyzed for age (A) and disease duration (B) at the time of spinal tap. Dots represent individual values for age (A) and duration (B), lines reflect median values. Statistics: Kruskal-Wallis test followed by Dunn’s multiple comparison test. P values of <0.05 were defined as statistically significant.

Moreover, we analyzed the correlation of age and disease duration with the antiviral immune response. We found a moderately higher age in patients with elevated AIs vs. those without (Figure 2A). Only a statistical trend was observed for disease duration, using the same type of analysis (Figure 2B). When looking at an arbitrary division at 5 years disease duration only 4 of 18 (22%) patients with less than 5 years had at least one elevated AI, while 17 of 28 (61%) patients with a disease duration of more than 5 years did so (Fisher’s exact test, p = 0.0156).

## Discussion

Here we report that an intrathecal, polyspecific antiviral immune response was detected in about one third of patients with relapsing-remitting and about two thirds of patients with chronic progressive MS, in whom no OCB were demonstrated in the CSF by isoelectric focusing and the standard IgG index was normal. In contrast, significantly fewer control patients showed an intrathecal antiviral immune response. While about one fourth of the MS patients had an elevated AI against at least two viruses, none of the control patients did so. The intrathecal, polyspecific antiviral immune response might therefore be useful as a differential diagnostic tool in OCB-negative patients, in whom MS is suspected. Previous studies had found the polyspecific antiviral immune response to be more specific for MS than OCB – highlighting its diagnostic value [1], [4]–[9].

The higher prevalence of antiviral antibodies in our chronic progressive MS patients in comparison to those with relapsing-remitting disease is of interest, as chronic progressive MS may diagnostically be particularly challenging. Different prevalence rates in our relapsing-remitting and our chronic progressive MS patients could be partially ascribed to age and disease duration, which both were moderately higher in patients with an antiviral immune response than in those without. This might also reflect a broadening of the immune reponse with increasing disease duration. Theoretically, different prevalence rates in older vs. younger patients may additionally be due to different antiviral vaccination states. Thirtytwo of the 46 MS patients were born before vaccinations against measles and rubella viruses were generally introduced in Germany in 1968 and 1969, respectively. Eleven of 12 MS patients with an elevated M AI and 10 of 11 MS patients with an elevated R AI were born before 1968. While any influence of vaccination programs on virus AIs observed here cannot be excluded the observation that also 8 of 10 patients with an elevated Z AI were born before 1968– long before the childhood vaccination program against varicella zoster was installed in 2004– argues against differences in vaccination states as an antigen-specific explanation of the observed age effect.

Median disease durations were comparable in PPMS and SPMS patients. However, our small subgroup of PPMS patients had a higher age at onset than our SPMS patients (p = 0.02) and male predominance in contrast to our SPMS patients (cf. Table 2), which might argue for two pathogenetically different groups of patients [14]. An antiviral immune response against at least two viruses was detected significantly more often in SPMS than in PPMS patients (cf. Table 3), and median AIs were overall higher in SPMS than in PPMS patients (data not shown). This might indicate a broader and stronger antiviral immune response in OCB-negative SPMS than PPMS patients, but the still small number of OCB-negative chronic progressive MS patients in our study limits the diagnostic validity until larger groups have been analysed.

We conclude that the intrathecal, polyspecific antiviral immune response might be considered as an additional laboratory-based diagnostic criterion for MS on top of OCB and the elevated IgG index. It may broaden the diagnostic armamentarium in establishing the diagnosis of MS in OCB-negative patients, once confirmed by other research groups in patients with definite MS. Furthermore, it might be worthwhile to evaluate its diagnostic usefulness in other populations, where the prevalence of OCB in the CSF is much lower than in Western countries [15].
